# The Dynamic Interplay of Lifestyle, Dietary Factors, and Cardiometabolic Risk in Hypertension: A Cross-Sectional Investigation Among Saudi Adults

**DOI:** 10.3390/diagnostics15162097

**Published:** 2025-08-20

**Authors:** Mohammad A. Jareebi

**Affiliations:** Department of Family and Community Medicine, Faculty of Medicine, Jazan University, Jazan 82816, Saudi Arabia; mjareebi@jazanu.edu.sa

**Keywords:** hypertension, nutrition, Saudi Arabia, lifestyle factors, obesity, smoking, family history, socioeconomic status, cardiometabolic comorbidities

## Abstract

**Background/Objectives**: Hypertension is a growing public health concern in Saudi Arabia, driven by rapid socioeconomic changes. This study investigated the interplay between habitual, behavioral, and dietary risk factors associated with hypertension among Saudi adults. **Methods**: A cross-sectional survey was conducted among 3312 Saudi adults using multistage stratified random sampling. The data were collected via validated questionnaires assessing sociodemographic, anthropometric indicators, lifestyle behaviors, dietary patterns, and medical history. Hypertension status was determined through self-reported diagnosis. Bivariate analyses and multiple logistic regression identified independent predictors (*p* < 0.05). **Results**: Hypertension prevalence was 13% (mean age: 34 ± 15 years; 50% male). The strongest predictors were age (OR = 1.08/year; 95% CI: 1.07–1.10; *p* < 0.001), increased body mass index (OR = 1.03; 95% CI: 1.01–1.06; *p* = 0.011), smoking (OR = 1.55; 95% CI: 1.04–2.29; *p* = 0.030), and family history of hypertension (OR = 7.71; 95% CI: 5.61–10.75; *p* < 0.001). Participants with diabetes mellitus had 89% higher odds of hypertension (OR = 1.89; 95% CI: 1.42–2.51; *p* < 0.001), and those with dyslipidemia had more than double the odds (OR = 2.45; 95% CI: 1.38–4.22; *p* = 0.002). Protective factors included higher income (≥15,000 SAR; OR = 0.54; 95% CI: 0.36–0.81; *p* = 0.003) and regular whole grain consumption (OR = 0.60; 95% CI: 0.46–0.77; *p* < 0.001). **Conclusions**: Hypertension risk in Saudi adults is shaped by age, obesity, smoking, comorbid metabolic conditions (diabetes and dyslipidemia), and genetic pre-disposition. In contrast, higher income and whole grain intake may offer protection. These findings underscore the need for comprehensive prevention strategies that address both lifestyle and cardiometabolic comorbidities, in alignment with Saudi Vision 2030 health priorities.

## 1. Introduction

Hypertension remains a critical public health challenge globally, affecting over 1.28 billion adults aged 30–79 worldwide, with two-thirds residing in low- and middle-income countries where nearly half remain unaware of their condition and fewer than one-fifth achieve adequate control [1,2]. In Saudi Arabia, a high-income nation undergoing rapid socioeconomic transformation, hypertension prevalence has escalated dramatically, with community-based studies reporting rates ranging from 15.2% to 32.6% [3,4].

This alarming trend coincides with widespread lifestyle changes including obesity rates exceeding 50% among adults, tobacco use, physical inactivity, dietary patterns dominated by high salt, sugar, and processed foods, and elevated stress levels compounded by sleep disturbances [5,6,7,8]. While international evidence confirms these lifestyle factors contribute significantly to hypertension and related cardiovascular outcomes [9,10,11,12,13,14], most Saudi studies have examined them in isolation rather than through integrated analytic frameworks that capture the synergistic effects of combined risk factors demonstrated in other populations.

Understanding the complex interplay between habitual behaviors (smoking, stress, sleep), behavioral patterns (physical activity, sedentary time), and dietary factors (sodium, sugar, processed foods) is biologically plausible given that physical inactivity can exacerbate pressor responses to high salt intake, stress increases cravings for high-calorie sodium-rich foods, and these combinations trigger sympathetic nervous system overactivation, endothelial dysfunction, and hormonal imbalances that elevate blood pressure [11,15,16,17].

This study therefore aims to assess the combined and individual associations of habitual, behavioral, and dietary risk domains with hypertension among Saudi adults aged ≥18 years using multivariate models to explore their interplay, providing integrated multidimensional evidence necessary for tailoring prevention strategies under Saudi Vision 2030 while contributing to global understanding of lifestyle interactions in hypertension risk for populations experiencing rapid urbanization and lifestyle transitions.

## 2. Materials and Methods

### 2.1. Study Design and Setting

This cross-sectional survey was conducted between May and June 2024 across multiple regions in Saudi Arabia to examine the determinants of hypertension among Saudi adults. The study employed a multistage stratified sampling approach targeting urban and rural populations throughout the Kingdom, ensuring representation of diverse socioeconomic strata and geographical regions, consistent with methodological approaches used in previous regional hypertension studies [3,4,18].

### 2.2. Study Population and Sampling

The target population comprised Saudi nationals aged 18 years and older residing in both urban and rural areas nationwide. Inclusion criteria were Saudi nationality, minimum age of 18 years, and provision of informed consent. Participants were excluded if pregnant, had severe chronic illnesses unrelated to hypertension or metabolic conditions, or were unable to complete assessments due to cognitive or physical impairments.

Sample size calculation was based on an estimated hypertension prevalence of 25% among Saudi adults [3,4], with 95% confidence level and 3% margin of error, accounting for design effect. The minimum required sample was approximately 3000 participants; to accommodate potential non-response and incomplete data, the final recruited sample included 3312 adults.

A multistage stratified random sampling method was implemented to ensure representativeness. First, regions were stratified by urban and rural characteristics. Second, clusters including universities, healthcare centers, and community centers were purposively selected within each stratum. Third, eligible adults were recruited within each cluster using systematic sampling methods. Sampling frames were derived from available census data and health registries where accessible, with random selection within each stratum to minimize sampling bias and enhance external validity.

### 2.3. Data Collection Procedures

Data collection employed structured, interviewer-administered questionnaires developed from validated instruments used in previous national health surveys and hypertension-related studies [18,19,20]. The questionnaires were available in both Arabic and English, with data collection supervised by trained medical and public health students who underwent standardized training protocols. A comprehensive pilot study was conducted among 50 participants to assess questionnaire clarity, cultural appropriateness, and relevance.

### 2.4. Measurements

#### 2.4.1. Sociodemographic Variables

Sociodemographic data included age (continuous), gender, educational attainment (postgraduate, university, secondary, and lower), marital status (single, married, divorced/widowed), monthly income in Saudi riyals (<SAR 5000 [<USD 1333], SAR 5000–9999 [USD 1333–USD 2666], SAR 10,000–14,999 [USD 2667–USD 3999], SAR ≥ 15,000 [USD ≥4000]), residence type (urban, rural), housing characteristics, and family size.

#### 2.4.2. Anthropometric Assessments

Height and weight measurements were reported by participants and body mass index (BMI) was calculated as weight in kilograms divided by height in meters squared and categorized according to WHO classifications: underweight (<18.5 kg/m^2^), normal weight (18.5–24.9 kg/m^2^), overweight (25.0–29.9 kg/m^2^), and obesity classes I–III (≥30.0 kg/m^2^) [21].

#### 2.4.3. Hypertension, Lifestyle, and Behavioral Variables

Hypertension status was determined through self-reported physician diagnosis.

Physical activity patterns were assessed using validated questions adapted from international surveys [22], categorized as no physical activity per week, moderate or vigorous activity for less than 30 min five times per week, or moderate or vigorous activity for minimum 30 min five times per week. Smoking status was classified as never smoker, current smoker, ex-smoker, or secondhand smoke exposure, following WHO STEPs methodology [19]. Sleep duration was recorded in hours per night, and participants rated their satisfaction with physical activity and body weight on 10-point scales.

#### 2.4.4. Dietary Assessment

Dietary patterns were evaluated through structured questions addressing consumption of whole grain products, fruits and vegetables (minimum 5 servings per day), low-fat meat choices, avoidance of high-sugar foods, and selection of low-fat products. Eating behavior satisfaction was assessed using a 10-point scale.

### 2.5. Statistical Analysis

Statistical analyses were performed using R software (version 4.2.3; R Foundation for Statistical Computing, Vienna, Austria). Descriptive statistics included means with standard deviations for continuous variables and frequencies with percentages for categorical variables. Normal distribution of continuous variables was assessed using Shapiro–Wilk tests and visual inspection of histograms.

Bivariate associations between hypertension status and participant characteristics were evaluated using appropriate statistical tests: independent t-tests for continuous variables and chi-square tests for categorical variables. Variables demonstrating significant associations (*p* < 0.05) in bivariate analyses were subsequently included in multivariate modeling.

Multiple logistic regression analysis was conducted to identify independent predictors of hypertension, with results presented as odds ratios (OR) with 95% confidence intervals (CI). Model assumptions including linearity of continuous predictors, absence of multicollinearity (variance inflation factor < 5), and adequate sample size were verified. Model fit was assessed using Tjur’s R-squared, and statistical significance was set at *p* < 0.05 throughout all analyses.

### 2.6. Ethical Considerations

Ethical approval was obtained from the Standing Committee for Scientific Research at Jazan University (IRB No. REC-45/05/848; dated 26 November 2023). The study adhered to the Declaration of Helsinki principles, with written informed consent obtained from all participants prior to enrollment. Participation was entirely voluntary and anonymous, with participants retaining the right to withdraw at any point without consequences. Data confidentiality and anonymity were strictly maintained throughout the study period, with secure data storage and access limited to authorized research personnel only.

### 2.7. Use of Generative Artificial Intelligence

Generative AI was used solely for language editing. Study design, data, analysis, and interpretation were entirely conducted by the author.

## 3. Results

### 3.1. Participant Characteristics

A total of 3312 Saudi adults participated in the study, with a mean age of 34 ± 15 years and balanced gender distribution (50% male, 50% female). The majority held university-level education (55%, n = 1830), while 41% (n = 1369) had secondary education or less, and 3% (n = 113) possessed postgraduate qualifications. Nearly half were single (49%, n = 1639), with 46% (n = 1540) married and 4% (n = 133) divorced or widowed. Income distribution revealed 31% (n = 1019) earning below SAR 5000 (USD < 1333) monthly, while 28% (n = 935) earned above SAR 15,000 (USD ≥ 4000). Most participants resided in rural areas (62%, n = 2067), with 47% living in owned villas (Table 1).

### 3.2. Anthropometric and Lifestyle Characteristics

Participants had a mean BMI of 25.0 ± 5.6 kg/m^2^, with 41% maintaining normal weight, 30% overweight, and 17% obese (classes I–III: 12%, 4%, 1%, respectively). Physical inactivity was prevalent, with 38% reporting no weekly exercise and only 21% reported following recommended activity guidelines of ≥30 min, five times weekly. The majority (81%) had never smoked, while 10% were current smokers. Mean sleep duration was 7.5 ± 1.8 h nightly, with moderate satisfaction scores for physical activity (5.2 ± 3.0) and body weight (6.0 ± 2.9) on 10-point scales (Table 2).

### 3.3. Dietary Patterns

**Figure 1:**
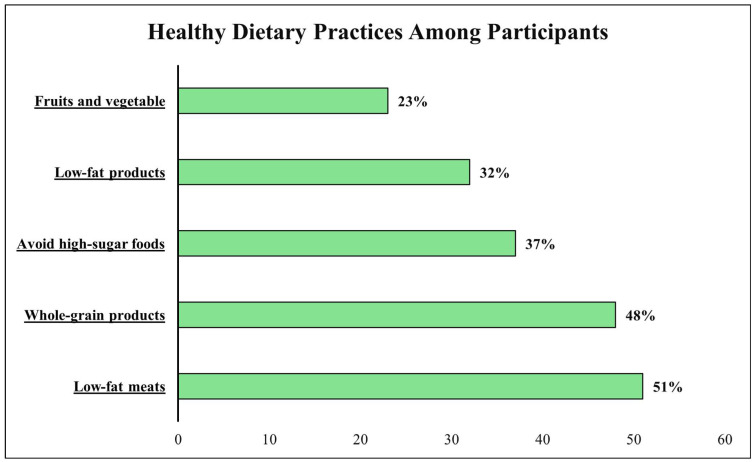
Dietary characteristics of participants (N = 3312).

Dietary assessment revealed suboptimal patterns across multiple domains. Less than half (48%, n = 1574) consumed whole grain products regularly, while only 23% (n = 747) achieved the recommended intake of five or more daily fruit and vegetable servings. Approximately half (51%, n = 1681) chose low-fat meat options, and only 32% (n = 1075) selected low-fat products overall. Sugar avoidance was practiced by 37% (n = 1212) of participants. Overall eating behavior satisfaction averaged 5.9 ± 2.6 on the 10-point scale (Figure 1).

### 3.4. Medical and Family History Profile

The family history of hypertension was highly prevalent (51%, n = 1692), followed by family history of cardiovascular disease (15%, n = 497). Clinically, 13% (n = 431) had physician-diagnosed hypertension, with an equal proportion (13%, n = 415) reporting diabetes mellitus. Dyslipidemia affected 3% (n = 108), while cardiovascular disease was present in 2% (n = 70) of participants (Table 3).

### 3.5. Hypertension Prevalence and Bivariate Associations

The overall prevalence of hypertension was 13% (n = 431) among the study population. Significant bivariate associations were observed across multiple domains. Hypertensive participants were significantly older (50 ± 15 vs. 31 ± 13 years, *p* < 0.001) and demonstrated distinct sociodemographic patterns, including different educational levels (*p* < 0.05), marital status distribution (*p* < 0.001), income categories (*p* < 0.001), and housing arrangements (*p* < 0.001).

Anthropometric differences were pronounced, with hypertensive individuals showing significantly higher BMI (28 ± 5.2 vs. 25 ± 5.5 kg/m^2^, *p* < 0.001), weight (73 ± 15 vs. 66 ± 17 kg, *p* < 0.001), and altered BMI category distribution (*p* < 0.001). Lifestyle factors demonstrated strong associations, with hypertensive participants reporting higher rates of physical inactivity (47% vs. 37%, *p* < 0.001), increased smoking prevalence (*p* < 0.001), and shorter sleep duration (7.1 ± 1.8 vs. 7.5 ± 1.8 h, *p* < 0.001).

Dietary patterns significantly differed between groups, with hypertensive participants showing a higher consumption of fruits and vegetables (28% vs. 22%, *p* < 0.001), increased selection of low-fat meats (63% vs. 49%, *p* < 0.001), and greater avoidance of high-sugar foods (46% vs. 35%, *p* < 0.001). Family history patterns were markedly different, with 84% of hypertensive participants reporting family history of hypertension compared to 46% in non-hypertensive individuals (*p* < 0.001). Similar patterns were observed for the family history of cardiovascular disease (*p* < 0.001) (Table 4).

### 3.6. Independent Predictors of Hypertension

Multiple logistic regression analysis (Table 5) identified several independent predictors of hypertension, with the model explaining 31% of the variance (Tjur’s R^2^ = 0.309). Age emerged as the strongest continuous predictor, with each additional year increasing hypertension odds by 8% (OR = 1.08; 95% CI: 1.07–1.10; *p* < 0.001).

Socioeconomic Determinants: Higher income demonstrated a protective effect, with participants earning SAR > 15,000 (≥USD 4000) monthly showing 46% lower odds of hypertension compared to the lowest income group (OR = 0.54; 95% CI: 0.36–0.81; *p* = 0.003). No significant associations were observed for education, marital status, residence type, or housing characteristics in the adjusted model.

Anthropometric and Lifestyle Factors: The BMI maintained significance as an independent predictor, with each unit increase associated with 3% higher odds (OR = 1.03; 95% CI: 1.01–1.06; *p* = 0.011). Current smoking increased hypertension odds by 55% compared to non-smokers (OR = 1.55; 95% CI: 1.04–2.29; *p* = 0.030). Physical activity levels and sleep duration did not retain significance in the multivariate model.

Dietary Factors: Dietary patterns showed mixed associations with hypertension risk. Regular whole grain consumption emerged as protective, reducing odds by 40% (OR = 0.60; 95% CI: 0.46–0.77; *p* < 0.001). Conversely, consuming ≥ 5 servings of fruits and vegetables daily was associated with 38% higher odds (OR = 1.38; 95% CI: 1.04–1.83; *p* = 0.025), while choosing low-fat meats increased odds by 37% (OR = 1.37; 95% CI: 1.06–1.78; *p* = 0.016). Sugar avoidance and low-fat product selection showed no significant associations in the adjusted model.

Family History Effects: Family history of hypertension was the strongest predictor overall, increasing odds more than sevenfold (OR = 7.71; 95% CI: 5.61–10.75; *p* < 0.001). This represents the most potent risk factor in the entire model, with the confidence interval entirely above the null value, confirming its robust association with hypertension risk.

Medical Comorbidities: Several diagnosed medical conditions showed significant independent associations with hypertension. Participants with diagnosed diabetes mellitus had 89% higher odds of hypertension (OR = 1.89; 95% CI: 1.42–2.51; *p* < 0.001), while those with diagnosed dyslipidemia showed more than doubled odds (OR = 2.45; 95% CI: 1.38–4.22; *p* = 0.002).

### 3.7. Summary of Key Findings

**Figure 2:**
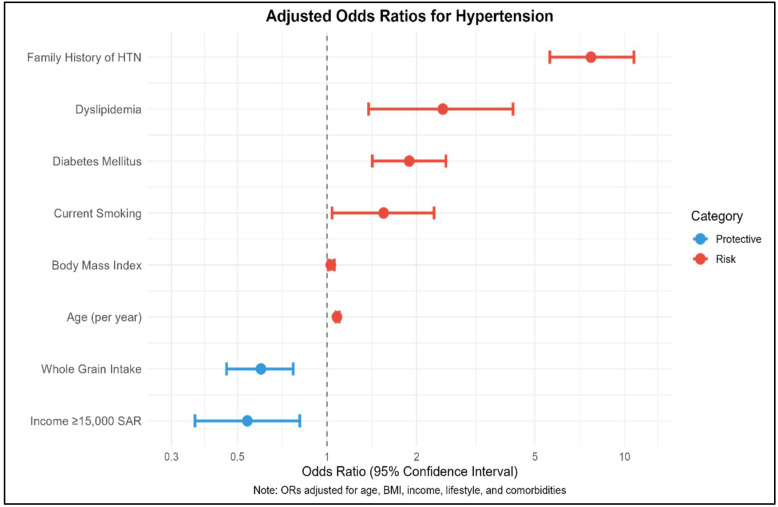
Key predictors of HTN risk.

The analysis revealed hypertension affects 13% of Saudi adults, with a complex interplay of modifiable and non-modifiable risk factors. Age, higher BMI, smoking, family history of hypertension, and medical comorbidities (diabetes mellitus, dyslipidemia, cardiovascular disease) emerged as primary risk factors, while higher income and whole grain consumption showed protective effects (Table 5 and Figure 2). The unexpected positive associations with healthy dietary choices (fruits/vegetables, low-fat meats) may reflect reverse causality, where hypertensive individuals adopt healthier eating patterns post-diagnosis. The model’s moderate explanatory power (R^2^ = 0.309) suggests additional unmeasured factors contribute to hypertension risk in this population.

## 4. Discussion

This comprehensive cross-sectional study of 3312 Saudi adults reveals hypertension prevalence of 13%, with complex interactions between demographic, lifestyle, and familial factors shaping cardiovascular risk. The findings provide critical insights into modifiable and non-modifiable determinants of hypertension in a Middle Eastern population undergoing rapid socioeconomic transition, with implications for both local intervention strategies and global understanding of lifestyle–hypertension relationships.

### 4.1. Age and Non-Modifiable Risk Factors

The strong association between advancing age and hypertension (OR = 1.08 per year; *p* < 0.001) aligns with established physiological mechanisms of vascular aging and corroborates findings from regional studies [3,4,18]. The China Health and Nutrition Survey (1991–2015) similarly documented rising age-standardized hypertension prevalence, confirming age as a universal risk marker [23]. While non-modifiable, age serves as a crucial identifier for targeting early preventive strategies to delay hypertension onset through lifestyle interventions [24].

### 4.2. Obesity as a Persistent Modifiable Risk

The significant association between BMI and hypertension (OR = 1.03 per unit; *p* = 0.011) reinforces obesity’s role as a primary modifiable risk factor, consistent with global meta-analyses showing 1.59–1.91 times higher hypertension risk among obese individuals across diverse populations [25]. This finding is consistent with national Saudi studies where BMI emerged as a strong predictor [26], and Al-Kharj research demonstrating nearly fivefold higher odds among overweight individuals [27]. The mechanistic pathway involves hyperinsulinemia, chronic inflammation, and sympathetic nervous system activation contributing to increased vascular resistance [28], highlighting the critical need for weight management interventions in Saudi Arabia where obesity affects over half the adult population [5].

### 4.3. Smoking and Cardiovascular Risk

Current smoking increased hypertension odds by 55% (OR = 1.55; *p* = 0.030), confirming tobacco use as an independent risk factor despite relatively low prevalence (10%) in our sample. This finding aligns with national Saudi surveys demonstrating significant smoking–hypertension associations after confounder adjustment and Riyadh studies showing threefold increases in uncontrolled hypertension risk among smokers [28,29]. International evidence from Chinese populations similarly reports higher ambulatory hypertension odds in smokers (OR = 1.69) [23], with nicotine-induced sympathetic activation and vascular injury providing mechanistic explanations [30]. Given national smoking rates reaching 17.8% [31], sustained tobacco control measures remain essential for hypertension prevention.

### 4.4. Socioeconomic Determinants and Health Equity

The protective effect of higher income (SAR > 15,000 monthly; OR = 0.54; *p* = 0.003) underscores socioeconomic status as a fundamental health determinant. Participants earning USD ≥ 4000 monthly demonstrated 46% lower hypertension odds compared to those earning USD < 1333, reflecting improved access to nutritious foods, healthcare services, and health-promoting environments [32]. This finding contrasts with some studies reporting higher income associated with increased hypertension risk due to sedentary lifestyles [33] but aligns with large Saudi studies documenting better hypertension awareness, treatment adherence, and control among higher-income groups [34]. The observed protective association between higher income and hypertension may be partially explained by better access to healthcare services, healthier dietary choices, and improved living conditions. However, unmeasured socioeconomic and environmental factors may also contribute, and residual confounding cannot be excluded. The socioeconomic gradient suggests that comprehensive hypertension prevention must address structural determinants alongside individual behavioral factors.

### 4.5. Dietary Patterns and Nutritional Paradoxes

Dietary findings reveal complex relationships, warranting careful interpretation. While whole grain consumption demonstrated protective effects (OR = 0.60; *p* < 0.001), consistent with established antihypertensive properties of dietary fiber, magnesium, and antioxidants [35], unexpected positive associations emerged for fruits/vegetables (OR = 1.38; *p* = 0.025) and low-fat meat consumption (OR = 1.37; *p* = 0.016). These counterintuitive findings likely reflect reverse causality, where hypertensive individuals adopt healthier eating patterns following diagnosis, contradicting established protective effects demonstrated in DASH trials [36] and meta-analyses emphasizing plant-rich diets [35]. The reliance on self-reported dietary patterns rather than validated quantitative assessments may have introduced reporting bias, highlighting the need for objective nutritional evaluation tools in future Saudi studies.

### 4.6. Family History and Genetic Susceptibility

Family history of hypertension emerged as the strongest independent predictor (OR = 7.71; *p* < 0.001), indicating powerful genetic or shared environmental influences. This sevenfold increase in odds exceeds many national [37] and international studies [38,39], suggesting particularly strong familial aggregation in Saudi populations. The unexpected protective association with family history of diabetes (OR = 0.66; *p* = 0.002) may reflect heightened health awareness and lifestyle modifications among metabolically vulnerable families [40], though residual confounding cannot be excluded. These findings emphasize the need for family-based prevention approaches and genetic counseling in high-risk Saudi families.

### 4.7. Lifestyle Factors and Behavioral Interventions

While physical inactivity, sleep disturbances, and weight dissatisfaction showed significant bivariate associations with hypertension, they lost significance in multivariate models, suggesting their effects may be mediated through other measured variables such as the BMI and age. Nevertheless, the strong bivariate association between physical inactivity and hypertension (*p* < 0.001) supports WHO recommendations for regular movement as a cornerstone of blood pressure control [19]. Meta-analyses confirm that regular moderate-to-vigorous physical activity reduces incident hypertension risk by up to 30% [41]. Physical activity remains an important target despite not achieving statistical significance in our adjusted model.

### 4.8. Clinical and Policy Implications

These findings have immediate relevance for Saudi Arabia’s Vision 2030 health objectives and broader global hypertension prevention efforts. The identification of age, BMI, smoking, income, and family history as key determinants supports multi-level intervention strategies combining individual behavioral counseling with structural policy changes. Healthcare systems should prioritize early screening among older adults and those with family histories, while implementing population-wide tobacco control, obesity prevention, and socioeconomic equity initiatives. The strong protective effect of higher income suggests that poverty reduction and healthcare access improvements may yield substantial cardiovascular benefits. Additionally, the complex dietary findings highlight the need for culturally tailored nutritional counseling that considers local food preferences and preparation methods.

### 4.9. Study Limitations and Future Directions

Several limitations warrant acknowledgment. The cross-sectional design precludes causal inference, particularly regarding dietary associations that may reflect reverse causality. Self-reported dietary and lifestyle data introduce potential recall and social desirability biases, while hypertension status was determined by self-reported physician diagnosis, which may introduce recall or reporting bias. Future studies should include standardized blood pressure measurements to enhance diagnostic accuracy. Although nationally representative, our sampling may not fully capture remote or institutionalized populations. The model’s moderate explanatory power (R^2^ = 0.309) suggests that unmeasured genetic, environmental, or behavioral factors likely contribute to hypertension risk. Despite adjusting for multiple covariates, residual confounding from unmeasured variables and potential measurement error from self-reported data may have attenuated some associations.

Future research should employ prospective designs with objective detailed dietary assessments, biomarker validation, and comprehensive genetic analyses to clarify familial contributions. Intervention studies evaluating culturally adapted lifestyle modification programs would provide critical evidence for clinical practice and policy development. Additionally, mechanistic studies exploring the protective association between family diabetes history and hypertension could yield novel insights into metabolic–cardiovascular interactions in Middle Eastern populations.

## 5. Conclusions

This study demonstrates that hypertension among Saudi adults results from complex interactions between demographic characteristics, socioeconomic factors, lifestyle behaviors, and genetic predisposition. While age and family history represent non-modifiable risks requiring targeted screening approaches, the significant associations with BMI, smoking, income, and dietary patterns offer multiple intervention opportunities. The findings support comprehensive prevention strategies that address both individual behaviors and structural determinants of health, aligning with Saudi Arabia’s Vision 2030 objectives while contributing to global evidence on lifestyle–hypertension relationships in transitioning populations. The unexpected dietary associations underscore the importance of longitudinal studies with objective assessments to guide evidence-based nutritional interventions for cardiovascular disease prevention in the Kingdom.

## Figures and Tables

**Table 1:** Sociodemographic characteristics of participants (N = 3312).

Variable	Mean ± SD/n (%)
**Age**	34 ± 15
**Gender**	
Female	1644 (50%)
Male	1668 (50%)
**Education**	
Postgraduate	113 (3%)
Secondary and lower	1369 (41%)
University	1830 (55%)
**Marital Status**	
Divorced/Widowed	133 (4%)
Married	1540 (46%)
Single	1639 (49%)
**Income (SAR)**	
<5000 (USD < 1333)	1019 (31%)
5000–9999 (USD 1333–USD 2666)	620 (19%)
10,000–14,999 (USD 2667–USD 3999)	738 (22%)
≥15,000 (USD ≥ 4000)	935 (28%)
**Residence**	
Rural	2067 (62%)
Urban	1245 (38%)
**Housing Type**	
Owned-apartment	671 (20%)
Owned-traditional house	727 (22%)
Owned-villa	1560 (47%)
Rented	354 (11%)
**Family Size**	7 ± 2.9

**Table 2:** Habitual and anthropometric characteristics of participants (N = 3312).

Variable	Mean ± SD/n (%)
**Height (cm)**	163 ± 9.2
**Weight (kg)**	67 ± 17
**BMI (kg/m^2^)**	25 ± 5.6
**BMI Categories**	
Underweight	374 (11%)
Normal weight	1366 (41%)
Overweight	995 (30%)
Obesity class I	414 (12%)
Obesity class II	121 (4%)
Obesity class III	42 (1%)
**Weight satisfaction (1–10)**	6 ± 2.9
**Physical activity**	
None	1268 (38%)
<30 min, 5 days/week	1341 (40%)
≥30 min, 5 days/week	703 (21%)
**Physical activity satisfaction (1–10)**	5.2 ± 3
**Smoking status**	
Never smoker	2684 (81%)
Current smoker	320 (10%)
Ex-smoker	144 (4%)
Second-hand smoker	164 (5%)
**Cigarette use**	282 (9%)
**Sleep duration (h)**	7.5 ± 1.8

**Table 3:** Family and medical history of participants (N = 3312).

Variable	Yes (%)
**Family history of hypertension**	51%
**Family history of cardiovascular disease (CVD)**	15%
**Diagnosed with dyslipidemia**	3%
**Diagnosed with diabetes mellitus (DM)**	13%
**Diagnosed with hypertension (HTN)**	13%
**Diagnosed with cardiovascular disease (CVD)**	2%

**Table 4:** Association between study variables and HTN (univariate analysis).

Variable	No HTN (n = 2881)	HTN (n = 431)	Test
**Age**	31 ± 13	50 ± 15	*p* < 0.001 ***
**Gender**			*p* = 0.90
Female	1431 (50%)	213 (49%)	
Male	1450 (50%)	218 (51%)	
**Education**			*p* < 0.05 *
Postgraduate	98 (3%)	15 (3%)	
Secondary and lower	1164 (40%)	205 (48%)	
University	1619 (56%)	211 (49%)	
**Social status**			*p* < 0.001 ***
Divorced/Widowed	88 (3%)	45 (10%)	
Married	1212 (42%)	328 (76%)	
Single	1581 (55%)	58 (13%)	
**Income**			*p* < 0.001 ***
SAR < 5000	912 (32%)	107 (25%)	
SAR 5000–9999	534 (19%)	86 (20%)	
SAR 10,000–14,999	614 (21%)	124 (29%)	
SAR ≥ 15,000	821 (28%)	114 (26%)	
**Residence**			*p* < 0.05 *
Rural	1778 (62%)	289 (67%)	
Urban	1103 (38%)	142 (33%)	
**BMI**	25 ± 5.5	28 ± 5.2	*p* < 0.001 ***
**BMI category**			*p* < 0.001 ***
Normal weight	1252 (43%)	114 (26%)	
Overweight	815 (28%)	180 (42%)	
Obesity class I	329 (11%)	85 (20%)	
Obesity class II	92 (3%)	29 (7%)	
Obesity class III	33 (1%)	9 (2%)	
Underweight	360 (12%)	14 (3%)	
**Physical activity**			*p* < 0.001 ***
None	1064 (37%)	204 (47%)	
<30 min, 5 days/week	1184 (41%)	157 (36%)	
≥30 min, 5 days/week	633 (22%)	70 (16%)	
**Smoking**			*p* < 0.001 ***
Never smoker	2362 (82%)	322 (75%)	
Current smoker	274 (10%)	46 (11%)	
Ex-smoker	99 (3%)	45 (10%)	
Second-hand smoker	146 (5%)	18 (4%)	
**Sleep hours**	7.5 ± 1.8	7.1 ± 1.8	*p* < 0.001 ***
**Whole grain consumption**			*p* = 0.96
Yes	1369 (48%)	205 (48%)	
No	1512 (52%)	226 (52%)	
**Fruits and vegetables ≥ 5/day**			*p* < 0.001 ***
Yes	625 (22%)	122 (28%)	
No	2256 (78%)	309 (72%)	
**Low-fat meat choice**			*p* < 0.001 ***
Yes	1410 (49%)	271 (63%)	
No	1471 (51%)	160 (37%)	
**Sugar avoidance**			*p* < 0.001 ***
Yes	1013 (35%)	199 (46%)	
No	1868 (65%)	232 (54%)	
**Family history of HTN**			*p* < 0.001 ***
Yes	1328 (46%)	364 (84%)	
No	1553 (54%)	67 (16%)	
**Family history of CVD**			*p* < 0.001 ***
Yes	410 (14%)	87 (20%)	
No	2471 (86%)	344 (80%)	

* Statistical significance markers: * *p* < 0.05; *** *p* < 0.001.

**Table 5:** Multivariate analysis of hypertension determinants: multiple logistic regression.

Predictors	Odds Ratios	95% CI	*p*-Value
**(Intercept)**	0.00	0.00–0.00	<0.001
**Age**	1.08	1.07–1.10	<0.001 ***
**Gender [Male]**	1.00	0.76–1.33	0.978
**Education**			
Secondary and lower	1.14	0.59–2.32	0.697
University	1.00	0.54–1.95	0.988
**Social status**			
Married	1.05	0.65–1.73	0.841
Single	0.81	0.42–1.57	0.534
**Income**			
5000–9999 SAR	0.80	0.54–1.18	0.269
10,000–14,999 SAR	1.02	0.70–1.49	0.906
≥15,000 SAR	0.54	0.36–0.81	0.003 **
**Residence [Urban]**	0.80	0.61–1.04	0.098
**Housing**			
Owned-traditional house	1.00	0.68–1.46	0.982
Owned-villa	0.91	0.66–1.27	0.588
Rented	1.04	0.63–1.68	0.882
**Family size**	1.03	0.98–1.07	0.244
**BMI**	1.03	1.01–1.06	0.011 *
**Physical activity**			
<30 min, 5 days/week	1.23	0.93–1.62	0.153
≥30 min, 5 days/week	1.08	0.76–1.54	0.657
**Cigarettes**	1.55	1.04–2.29	0.030 *
**Sleep hours**	0.96	0.89–1.04	0.309
**Whole grain consumption**	0.60	0.46–0.77	<0.001 ***
**Fruits and vegetables ≥ 5/day**	1.38	1.04–1.83	0.025 *
**Low-fat meat choice**	1.37	1.06–1.78	0.016 *
**Sugar avoidance**	1.08	0.83–1.39	0.564
**Low-fat products**	1.08	0.83–1.41	0.547
**Family history of HTN**	7.71	5.61–10.75	<0.001 ***
**Dyslipidemia**	2.45	1.38–4.22	0.002 **
**Diabetes mellitus**	1.89	1.42–2.51	<0.001 ***
**Observations**	3312		
**R^2^ Tjur**	0.309		

* Statistical significance markers: * *p* < 0.05; ** *p* < 0.01; *** *p* < 0.001.

## Data Availability

The data supporting the findings of this study are available from the corresponding author upon reasonable request.

## Abbreviations

The following abbreviations are used in this manuscript:

HTN	Hypertension
CVD	Cardiovascular Disease
BMI	Body Mass Index
OR	Odds Ratio
CI	Confidence Interval
R2	Coefficient of Determination
SAR	Saudi Riyal

## References

[1] World Health Organization (2023). Hypertension Fact Sheet. https://www.who.int/news-room/fact-sheets/detail/hypertension.

[2] Mills K.T., Stefanescu A., He J. (2020). The global epidemiology of hypertension. Nat. Rev. Nephrol..

[3] Alshammari S.A., Alshammari A.S., Alshammari H.S., Ahamed S.S. (2023). Hypertension in Saudi Arabia: Prevalence, risk factors, and prevention strategies. Saudi Med. J..

[4] Al-Nozha M.M., Abdullah M., Arafah M.R., Khalil M.Z., Khan N.B., Al-Mazrou Y.Y., Al-Maatouq M.A., Al-Marzouki K., Al-Khadra A.H., Nouh M.S. (2007). Hypertension in Saudi Arabia. Saudi Med. J..

[5] Alqarni S.S.M. (2016). A review of prevalence of obesity in Saudi Arabia. J. Obes. Eat. Disord..

[6] Bassiony M.M. (2009). Smoking in Saudi Arabia. Saudi Med. J..

[7] Al-Hazzaa H.M. (2004). Physical inactivity in Saudi Arabia: An urgent public health concern. Saudi Med. J..

[8] Al-Hazzaa H.M., Musaiger A.O. (2011). Physical activity patterns and eating habits of adolescents living in major Arab cities. Arab. J. Food Nutr..

[9] Knutson K.L., von Schantz M. (2018). Associations between chronotype, morbidity and mortality in the UK Biobank cohort. Chronobiol. Int..

[10] Gangwisch J.E., Heymsfield S.B., Boden-Albala B., Buijs R.M., Kreier F., Pickering T.G., Rundle A.G., Zammit G.K., Malaspina D. (2006). Short sleep duration as a risk factor for hypertension: Analyses of the first National Health and Nutrition Examination Survey. Hypertension.

[11] NCD Risk Factor Collaboration (NCD-RisC) (2017). Worldwide trends in blood pressure from 1975 to 2015: A pooled analysis of 1479 population-based measurement studies with 19.1 million participants. Lancet.

[12] Mozaffarian D., Appel L.J., Van Horn L. (2011). Components of a cardioprotective diet: New insights. Circulation.

[13] Appel L.J., Moore T.J., Obarzanek E., Vollmer W.M., Svetkey L.P., Sacks F.M., Bray G.A., Vogt T.M., Cutler J.A., Windhauser M.M. (1997). A clinical trial of the effects of dietary patterns on blood pressure. N. Engl. J. Med..

[14] Huang Y., Cai X., Mai W., Li M., Hu Y. (2013). Association between prehypertension and cardiovascular outcomes: A systematic review and meta-analysis of prospective studies. Curr. Hypertens. Rep..

[15] He F.J., MacGregor G.A. (2007). Salt, blood pressure and cardiovascular disease. Curr. Opin. Cardiol..

[16] Oliver G., Wardle J., Gibson E.L. (2000). Stress and food choice: A laboratory study. Psychosom. Med..

[17] Grassi G., Seravalle G., Quarti-Trevano F., Dell’Oro R., Arenare F., Tana F., Bolla G., Mancia G. (2009). Sympathetic and baroreflex cardiovascular control in hypertension-related left ventricular dysfunction. Hypertension.

[18] Al-Thani M., Al-Thani A.A., Al-Chetachi W., Khalifa S.A.H., Al Malki B., Khalid H., Taha Z. (2016). Prevalence and determinants of metabolic syndrome and its components among Qatari adults: A cross-sectional study. Diabetes Metab. Syndr..

[19] World Health Organization (2017). WHO STEPwise Approach to NCD Risk Factor Surveillance (STEPS).

[20] Whelton P.K., Carey R.M., Aronow W.S., Casey D.E., Collins K.J., Dennison Himmelfarb C., DePalma S.M., Gidding S., Jamerson K.A., Jones D.W. (2018). 2017 ACC/AHA/AAPA/ABC/ACPM/AGS/APhA/ASH/ASPC/NMA/PCNA Guideline for the Prevention, Detection, Evaluation, and Management of High Blood Pressure in Adults. J. Am. Coll. Cardiol..

[21] World Health Organization (2000). Obesity: Preventing and Managing the Global Epidemic.

[22] Bull F.C., Al-Ansari S.S., Biddle S., Borodulin K., Buman M.P., Cardon G., Carty C., Chaput J.P., Chastin S., Chou R. (2020). World Health Organization 2020 guidelines on physical activity and sedentary behaviour. Br. J. Sports Med..

[23] Ma S., Yang L., Zhao M., Magnussen C.G., Xi B. (2021). Trends in hypertension prevalence, awareness, treatment and control rates among Chinese adults, 1991–2015. J. Hypertens..

[24] Budreviciute A., Damiati S., Sabir D.K., Onder K., Schuller-Goetzburg P., Plakys G., Katileviciute A., Khoja S., Kodzius R. (2020). Management and prevention strategies for non-communicable diseases (NCDs) and their risk factors. Front. Public Health.

[25] Khoja A., Andraweera P.H., Lassi Z.S., Padhani Z.A., Ali A., Zheng M., Pathirana M.M., Aldridge E., Wittwer M.R., Chaudhuri D.D. (2024). Modifiable and non-modifiable risk factors for premature coronary heart disease (PCHD): Systematic review and meta-analysis. Heart Lung Circ..

[26] Almajwal A.M., Al-Baghli N.A., Batterham M.J., Williams P.G., Al-Turki K.A., Al-Ghamdi A.J. (2009). Performance of body mass index in predicting diabetes and hypertension in the Eastern Province of Saudi Arabia. Ann. Saudi Med..

[27] Aldiab A., Shubair M.M., Al-Zahrani J.M., Aldossari K.K., Al-Ghamdi S., Househ M., Razzak H.A., El-Metwally A., Jradi H. (2018). Prevalence of hypertension and prehypertension and its associated cardioembolic risk factors; A population-based cross-sectional study in Alkharj, Saudi Arabia. BMC Public Health.

[28] AlMarri E.A., Al-Hamad J. (2020). Prevalence of obesity among hypertensive patients in Primary Care Clinic, Security Forces Hospital, Riyadh, Saudi Arabia 2017–2018: A prospective cross-sectional study. J. Fam. Med. Prim. Care.

[29] Prevalence of Risk Factors of Essential Hypertension among Saudis in Riyadh City. *IJMRHS*. https://www.ijmrhs.com/medical-research/prevalence-of-risk-factors-of-essential-hypertension-among-saudis-in-riyadh-city-74359.html.

[30] Haass M., Kübler W. (1997). Nicotine and sympathetic neurotransmission. Cardiovasc. Drugs Ther..

[31] Global Action to End Smoking Saudi Arabia—Tobacco Around the World. https://globalactiontoendsmoking.org/research/tobacco-around-the-world/saudi-arabia/.

[32] Cui X., Chang C.T. (2021). How income influences health: Decomposition based on absolute income and relative income effects. Int. J. Environ. Res. Public Health.

[33] Mugisha E.K. (2024). Hypertension and its correlation with socioeconomic status in East African urban and rural populations: A scientific review. Newport Int. J. Public Health Pharm..

[34] Saeed A.A., Al-Hamdan N.A., Bahnassy A.A., Abdalla A.M., Abbas M.A., Abuzaid L.Z. (2011). Prevalence, awareness, treatment, and control of hypertension among Saudi adult population: A national survey. Int. J. Hypertens..

[35] Streppel M.T., Arends L.R., van ’t Veer P., Grobbee D.E., Geleijnse J.M. (2005). Dietary fiber and blood pressure: A meta-analysis of randomized placebo-controlled trials. Arch. Intern. Med..

[36] Filippou C.D., Tsioufis C.P., Thomopoulos C.G., Mihas C.C., Dimitriadis K.S., Sotiropoulou L.I., Chrysochoou C.A., Nihoyannopoulos P.I., Tousoulis D.M. (2020). Dietary Approaches to Stop Hypertension (DASH) diet and blood pressure reduction in adults with and without hypertension: A systematic review and meta-analysis of randomized controlled trials. Adv. Nutr..

[37] Yagoub U., Saiyed N.S., Qahtani B.A., Al Zahrani A.M., Birema Y., Hariri I.A. (2022). Investigating the incidence and risk factors of hypertension: A multicentre retrospective cohort study in Tabuk, Saudi Arabia. PLoS ONE.

[38] Ranasinghe P., Cooray D.N., Jayawardena R., Katulanda P. (2015). The influence of family history of hypertension on disease prevalence and associated metabolic risk factors among Sri Lankan adults. BMC Public Health.

[39] Zare M.G., Okati-Aliabad H., Ansari-Moghaddam A., Mohammadi M., Shahraki-Sanavi F. (2023). Prevalence and risk factors of pre-hypertension and hypertension among adults in Southeastern Iran: Findings from the baseline survey of the Zahedan adult cohort study. PLoS ONE.

[40] Jabbar J.A., Al Masri A.H., Oweidat F.A., Al Ahmad M.M., Ali O.B., Oweidat Z.A., Sreedharan J. (2023). Family history of diabetes, hypertension, obesity and cardiovascular diseases in relation to self-health-care. Int. J. Community Med. Public Health.

[41] Pescatello L.S., Buchner D.M., Jakicic J.M., Powell K.E., Kraus W.E., Bloodgood B., Campbell W.W., Dietz S., Dipietro L., George S.M. (2019). Physical activity to prevent and treat hypertension: A systematic review. Med. Sci. Sports Exerc..

